# Attention-Related Eye Vergence Measured in Children with Attention Deficit Hyperactivity Disorder

**DOI:** 10.1371/journal.pone.0145281

**Published:** 2015-12-22

**Authors:** Maria Solé Puig, Laura Pérez Zapata, Laura Puigcerver, Neus Esperalba Iglesias, Carmen Sanchez Garcia, August Romeo, Josep Cañete Crespillo, Hans Supèr

**Affiliations:** 1 Dept Basic Psychology, Faculty of Psychology, University of Barcelona (UB), Barcelona, Spain; 2 Hospital Sant Joan de Deu (HSJD), Barcelona, Spain; 3 Mental Health Dept, Consorci Sanitari del Maresme (CSdM), Mataró, Spain; 4 Institute for Brain, Cognition and Behavior (IR3C), Barcelona, Spain; 5 Catalan Institution for Research and Advanced Studies (ICREA), Barcelona, Spain; Bournemouth University, UNITED KINGDOM

## Abstract

Recent evidence shows a novel role for eye vergence in orienting attention in adult subjects. Here we investigated whether such modulation in eye vergence by attention is present in children and whether it is altered in children with ADHD compared to control subjects. We therefore measured the angle of eye vergence in children previously diagnosed with ADHD while performing a cue task and compared the results to those from age-matched controls. We observed a strong modulation in the angle of vergence in the control group and a weak modulation in the ADHD group. In addition, in the control group the modulation in eye vergence was different between the informative cue and uninformative cue condition. This difference was less noticeable in the ADHD group. Our study supports the observation of deficient binocular vision in ADHD children. We argue that the observed disruption in vergence modulation in ADHD children is manifest of altered cognitive processing of sensory information. Our work may provide new insights into attention disorders, like ADHD.

## Introduction

Attention deficit hyperactivity disorder (ADHD) is one of the most common neurodevelopmental disorders affecting an estimated 3 to 7% of school-aged children worldwide. It is characterized by a low degree of attention, a high degree of hyperactivity and impulsivity, and the inability to inhibit inappropriate actions.

Several studies show that subjects with attention disorders like ADHD show atypical oculomotor behavior. In particular, compared to controls ADHD patients show an increased variability of saccadic responses [1,2]. Increased variability in ADHD patients has been observed in anti-saccades [1], visually guided saccades [1,3,4], and memory guided saccades [5]. Recent studies found that micro-saccades, rather than being randomly distributed, had directions that were directly correlated with the directions of covert attention shifts [6] and that the average rate of micro-saccades modulated (both suppression and enhancement) after cue presentation [7]. In ADHD subjects, micro-saccade rate is higher compared to control subjects, especially in the time interval around stimulus onset [8].

**Fig 1:**
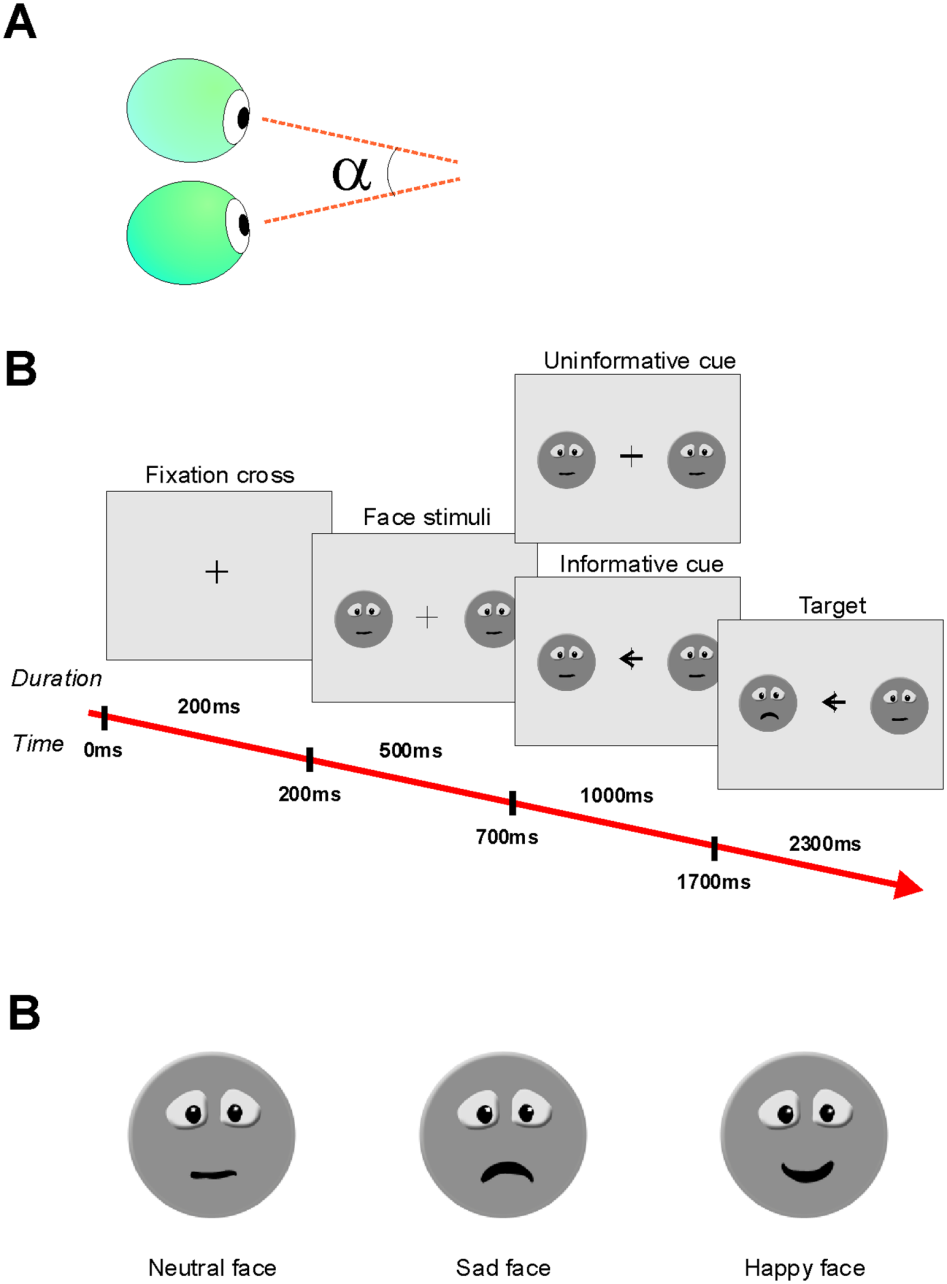
Schematic explanation of the vergence and task design. **A**: The eyes focus on a single point in space where the angle of vergence (alpha) relates to the distance of the focus point to the eyes. **B**: Illustration of the task and stimulus presentation times. **C**: Face images use in the task.

Vergence refers to the simultaneous movement of both eyes in opposite directions to obtain single binocular vision (Fig 1A). Precise binocular coordination however does not always happen and disparity is frequently observed during gaze fixation [9]. Fixation disparity is classified as either crossed (where the left eye fixates further to the right than the right eye) or uncrossed (where the right eye fixates further to the right than the left eye), which is more prevalent during the majority of fixations [10]. During gaze fixation, the eyes slowly convergence as fixation disparity at fixation onset is more divergent than at fixation offset. Convergence eye movement during fixation may derive from the fact that the two eyes typically become diverged during a saccade and become aligned during fixation [11]. Disparity has been observed in reading studies, e.g. [12–14]. Several reading studies have investigated whether processing difficulty influences binocular coordination [15–16]. The atypical eye movement’s patterns observed in dyslexic children suggest a deficiency in the visual attention processing as well as an immaturity of the ocular motor saccade and vergence systems interaction [17].

Recently we found a relationship between eye vergence and orienting visuospatial attention in human adults [18]. We observed that during gaze fixation the eyes briefly converge after the presentation of a stimulus that indicated the location of an upcoming visual target but not or weakly after a stimulus that was not informative about the location of the target [18]. Moreover detected but not unnoticed targets were accompanied by eye vergence. These results demonstrate that during covert shifts of attention the eyes briefly converge in adults.

Here we tested whether attention-related eye vergence also occurs in children. As poor binocular coordination in children is linked to ADHD [19] we also tested whether eye vergence is different in children with ADHD. We therefore measured the modulation of the angle of vergence in children previously diagnosed with ADHD while performing a cue task and compared the results to age-matched controls. In the task subjects shift their attention only when the target location is cued. In control subjects and to a lesser extent in ADHD subjects we expect to see the eyes to converge during fixation when they orient attention. As medical treatment reduces ADHD symptoms we also compared vergence responses from medicated children to the ones from non-medicated children.

## Methods

### Participants

Twenty-nine children diagnosed with ADHD, aged between 7 and 14 years (mean = 10.6 years; SD = 2.5), and a similar number (thirty-five) of sex/age matched healthy children participated in the experiment. Children with ADHD were classified as type Combined (N = 10), as Inattentive (N = 4) or as Hyperactive (N = 15). Twenty participants of the ADHD group were taking stimulant medication (Methylphenidate). The ADHD diagnosed children were all recruited through the Child and Adolescent Health Mental Center from Consorci Sanitari del Mareme, a public health consortium in Maresme (a nearly 400.000 inhabitant’s area in the North of Barcelona). Cases were diagnosed using the American Psychiatric Association's Diagnostic and Statistical Manual-IV-Text Revision criteria including a psychiatric and psychologist interview to assess the presence of symptoms of in-attention, hyperactivity and impulsivity during the last 6 months. Also the beginning of the symptoms before 7 years of age and the persistence of clinical dysfunction in at least two settings (school and home) were used as criteria [20]. Reasons for exclusion were mental retardation, other neurologic or mental disorder, or ophthalmologic problems. Part of the medical examination and psychiatric evaluation of all patients for diagnosing ADHD was the inquiry about visual problems. The survey included specific questions on strabismus and accommodation insufficiency. Patients with visual problems were discarded from the study. The children from the control group were recruited via two public schools showing no attention or conduct problems. All participants had normal or corrected-to-normal visual acuity.

### Ethics statement

The children were given detailed instructions for the experiments. Before participating in the study written informed consent from the parents on behalf of the children enrolled in our study was obtained in accordance with the Helsinki Declaration. The study was approved by the Ethics Committee of the University of Barcelona and of Consorci Sanitari del Maresme.

### Apparatus

We used EventIDE (Okazolab Ltd, London, UK) for presenting the visual stimuli. The display resolution was 1024 x 768 pixels. The participants’ position of gaze was monitored using a binocular remote eye-tracking system at a sample rate of 120 Hz (T120, Tobii Technology AB, Sweden).

### Procedure

Participants sat in a dimly lit room of the hospital or schools, in front of the PC monitor at a distance of 40–60 cm. To have similar conditions lights were switched off and curtains closed. The eye tracker was positioned in such a way that ambient lighting did not affect the recordings. The eye tracking equipment was calibrated (9 points, monocular) for each participant at the beginning of the experiment. Gaze fixations of at least 1000 ms within a region of 2°-3° around each calibration point were considered accurate. As subject needed only to fixate centrally during the task, mapping gaze position was secondary. Before starting the task, all participants practiced with some cue and no-cue trials (typically around 10–20 trials) to become familiar with the task. In both groups (ADHD and control) a similar number of practice trials were offered. When they confirmed that they understood the task we ran the experiment.

### Visual cue experiment

To assess orienting visual attention we used a paradigm in which participants were required to discriminate the emotion (happy/sad) of a cartoon face placed in the periphery. During the task children had to maintain fixation at a central cross. On half of the trials participants were informed of the location of the face stimulus by a central cue stimulus (informative cue condition). On the other half they were unaware of the target location (uninformative cue condition).

The experiment (Fig 1B) consisted of 4 sets with 32 trials each (128 trials in total) with 2 or 4 face images as possible targets. The 4 faces condition was introduced to make the task more difficult and was applied to the children older than 12 years of age. After eye calibration, observers were required to fixate a central cross with a size 0.7°x0.7° in the 2 face condition and 0.54°x0.54° in the 4 face condition. After 500 ms, 2 or 4 neutral face images (size of 4.1°x4.1° in the 2 face condition and 3.5°x3.5° in the 4 face condition) appeared in the periphery at an eccentricity of 4.8° or 6.4° for 2 or 4 faces, respectively. In the 2 face condition the faces were located on the left and right side of the fixation spot (Fig 1B). In the 4 face condition they were distributed, at an equidistant from each other, on an imaginary circle at angles of 45°, 135°, 225°, 315°.

After 1000 ms, an informative cue stimulus or an uninformative cue stimulus appeared on top of the fixation cross. In both tasks the informative cue was an arrowhead with a size of 0.32° or 0.35° for 2 and 4 faces, respectively. The uninformative cue stimulus was a central cross with a size of 1.4°x1.4°. The informative cue was always valid. After an additional period of 1000 ms, one of the peripheral faces changed into a happy or sad face (= target; Fig 1C). In the 2 face condition the emotion of the face disappeared at the end of the trial, i.e. response time while in the 4 face condition, the face emotion disappeared after 300ms to make that task more demanding. Participants had to respond by pressing a button to indicate whether the face was happy or sad. Feedback was not given to the observers. After the end of the trial a new one started automatically.

### Data analysis

We calculated the angle of vergence by transforming the eye recordings (*X* and *Y* coordinates of both eyes) into angular units through algorithms designed to calculate 3-D components (Sx, Sy, Sz) of both eye gaze vectors. Sx,y,z are the three coordinates which represent the intersection point of the lines describing the line of sight of each eye. When these lines do not intersect this represents the midpoint of the smallest distance between them. The transformation was performed using a distance of the screen to the observer (DSD) of 50 cm and 5.5 cm for the inter-pupil distance (IPD). The angle of vergence is the angle at which the intersection of both eye gaze vectors made the least error. Sx,y,z are calculated as follow:

$$S_{x}=\frac{{\text{IPD(IPD}}^{2}({\text{Y}}_{\text{l}}{\text{-Y}}_{\text{r}})({\text{Y}}_{\text{l}}+{\text{Y}}_{\text{r}})+\text{4IPD}({\text{-X}}_{\text{r}}{\text{Y}}_{l}^{2}{\text{-X}}_{\text{l}}{\text{Y}}_{\text{r}}^{2}-({\text{X}}_{\text{r}}+{\text{X}}_{\text{l}}){\text{DSD}}^{2})+\text{4}({\text{X}}_{\text{r}}^{2}({\text{Y}}_{l}^{2}+{\text{DSD}}^{2}){\text{-X}}_{l}^{2}({\text{Y}}_{\text{r}}^{2}+{\text{DSD}}^{2})))}{2{\left({\text{-2X}}_{\text{r}}{\text{Y}}_{\text{l}}+{\text{2X}}_{\text{l}}{\text{Y}}_{\text{r}}+\text{IPD}({\text{Y}}_{\text{l}}+{\text{Y}}_{\text{r}})\right)}^{2}+\;8({\text{(IPD}+{\text{X}}_{\text{l}}{\text{-X}}_{\text{r}})}^{2}+{\left({\text{Y}}_{\text{l}}{\text{-Y}}_{\text{r}}\right)}^{2}){\text{DSD}}^{2}}$$ (1)

$$S_{y}=\frac{{\text{2IPD(Y}}_{\text{l}}{\text{Y}}_{\text{r}}(-2{\text{X}}_{\text{l}}{\text{Y}}_{\text{r}}+{\text{2X}}_{\text{l}}{\text{Y}}_{\text{r}}^{}+{\text{IPD(Y}}_{\text{l}}+{\text{Y}}_{\text{r}}^{}))+(IPD+{\text{X}}_{\text{l}}-{\text{X}}_{\text{r}})({\text{Y}}_{\text{l}}^{}+{\text{Y}}_{\text{r}}^{}){\text{DSD}}^{2})}{{\left({\text{-2X}}_{\text{r}}{\text{Y}}_{\text{l}}+{\text{2X}}_{\text{l}}{\text{Y}}_{\text{r}}+\text{IPD}({\text{Y}}_{\text{l}}+{\text{Y}}_{\text{r}})\right)}^{2}+\;4({\text{(IPD}+{\text{X}}_{\text{l}}{\text{-X}}_{\text{r}})}^{2}+{\left({\text{Y}}_{\text{l}}{\text{-Y}}_{\text{r}}\right)}^{2}){\text{DSD}}^{2}}$$ (2)

$$S_{z}=\frac{\text{IPD}({\text{Y}}_{\text{l}}+{\text{Y}}_{\text{r}})({\text{-2X}}_{\text{r}}{\text{Y}}_{\text{l}}+{\text{2X}}_{\text{l}}{\text{Y}}_{\text{r}}+{\text{IPD(Y}}_{\text{l}}+{\text{Y}}_{\text{r}}))\text{DSD}+\text{4IPD(IPD}+{\text{X}}_{\text{l}}{\text{-X}}_{\text{r}}){\text{DSD}}^{\text{3}}}{{\left({\text{-2X}}_{\text{r}}{\text{Y}}_{\text{l}}+{\text{2X}}_{\text{l}}{\text{Y}}_{\text{r}}+{\text{IPD(Y}}_{\text{l}}+{\text{Y}}_{\text{r}})\right)}^{2}+\text{4(}{\left({\text{IPD+X}}_{\text{l}}{\text{-X}}_{\text{r}}\right)}^{2}+{\left({\text{Y}}_{1}{\text{-Y}}_{\text{r}}\right)}^{2}){\text{DSD}}^{2}}$$ (3)

From these components we calculated the saccade amplitude by Eq (4) azimuth (horizontal component of the eye movement), Eq (5) latitude (vertical component of the eye movement) and the focalized distance by Eq (6) vergence angles:

$$Azimuth\left(\alpha \right)=\text{arctan}\left(\frac{S_{x}}{S_{z}}\right)$$ (4)

$$Latitude\left(\alpha \right)=\text{arctan}\left(\frac{S_{y}}{\sqrt{S_{x}^{2}+S_{z}^{2}}}\right)$$ (5)

$$Vergence\left(\beta \right)=arctg\left(\frac{DIP/2}{\left\|S\right\|}\right)$$ (6)

Where ||*S*|| was calculated as:

$$\left\|S\right\|=\sqrt{S_{x}^{2}+S_{y}^{2}+S_{z}^{2}}$$ (7)

Only correct trials were analyzed. Trials with blinks and in which the subject made a saccadic eye movement in the relevant data segment were filtered out. In total there were 4120 trials in the control group and 3112 trials in ADHD group. The final data set consisted of 884 (43%) cue and 1002 (47%) no-cue trials in the control group and 723 (46%) cue and 829 (53%) no-cue trials in the ADHD group. From the eye position data, saccades were detected by using a vector velocity threshold of approximately 200 degrees per second. For statistical analysis t-tests and a multi-comparison ANOVA test were used.

## Results

### Behavior

Average detection performance of the ADHD group (89% and 87% for the targets in the informative and uninformative cue condition, respectively) was similar (informative cue condition: t-test_28_ = 1.40, p = 0.17; uninformative cue condition: t-test_28_ = 1.88, p = 0.07) to the detection performance of the control group (informative cue condition: 93%; uninformative cue condition: 93%). No significant difference in detection performance (Control group: t-test_13_ = -0.26, p = 0.79; ADHD group: t-test_12_ = 0.03, p = 0.97) and reaction time (Control group: t-test_13_ = -0.25, p = 0.80; ADHD group: t-test_12_ = 0.44, p = 0.60) was observed between the 2 and 4 faces condition. Neither differences between informative cue and uninformative cue conditions in detection performance were observed (Control group: paired t-test_28_ = -0-06, p = 0.95; ADHD group: paired t-test_28_ = 0.57, p = 0.57). The behavioral reaction times of the informative cue trials and uninformative cue trials (mean±sem; Control group: 789.4ms±32.7ms vs. 944.3±45.2ms; ADHD group: 861±36.6 vs. 967.1±43.7ms) were different (Control group: paired t-test_25_ = -4.16, p<10^−5^; ADHD group: paired t-test_27_ = -3.74, p<10^−5^). No differences in reaction times were observed between the control and ADHD group (informative cue condition: test_20_ = -1.18; p = 0.24; uninformative cue condition: test_20_ = -0.61, p = 0.55).

### Eye data

**Fig 2:**
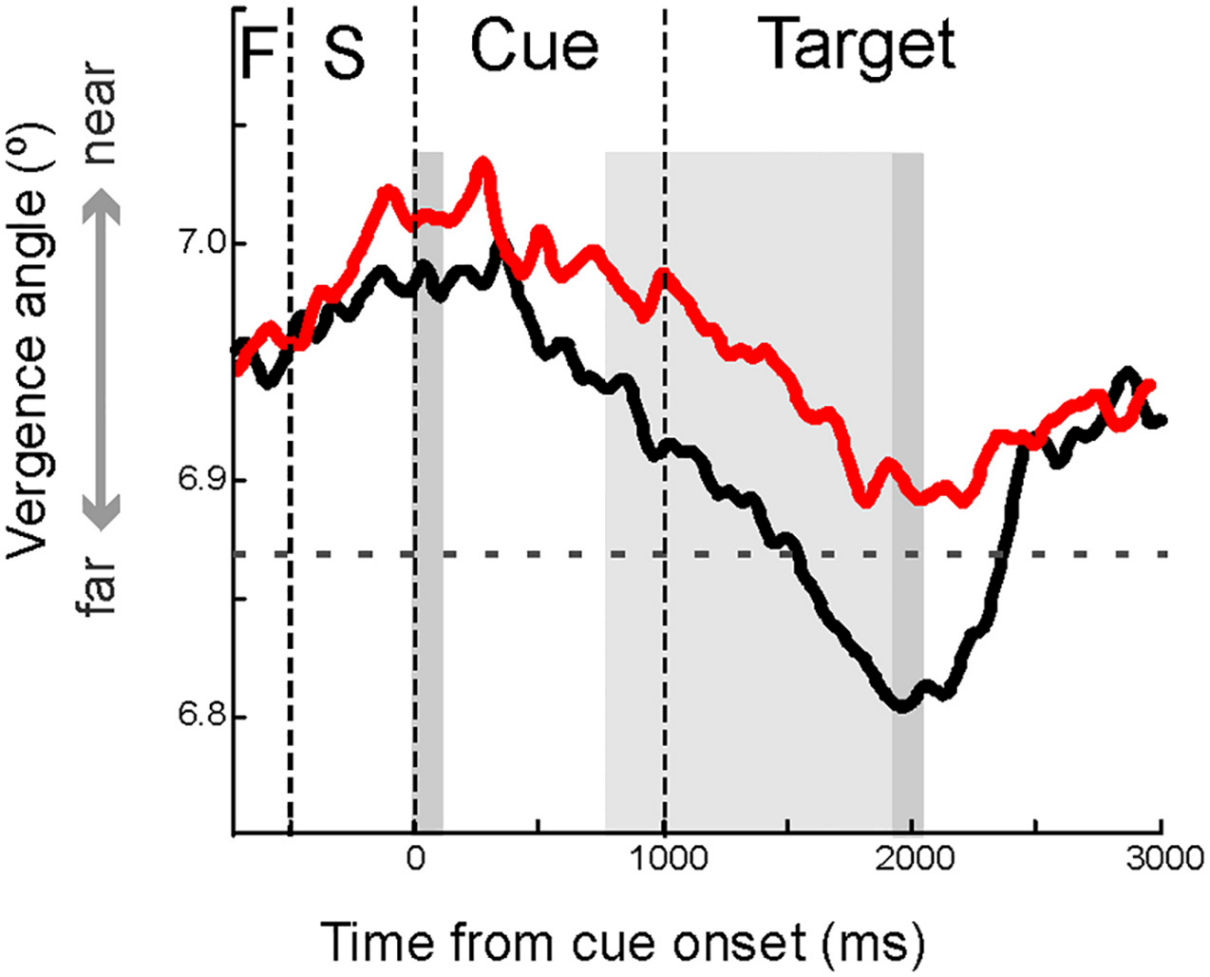
Average modulation across all conditions (cue and no-cue) of the angle of vergence during the time of a trial. Black/red trace denotes average angle of eye vergence from control/ADHD subjects. The grey shaded bars indicate the time periods for data analysis as explained in the main text. Phases in the task are demarcated by vertical dashed lines (F denotes fixation period and S the stimulus period). Horizontal dashed line indicates vergence angle belonging to the estimated screen distance.

Fig 2 shows the modulation in the angle of eye vergence throughout the trial both for the control and for the ADHD group. It can be seen that eye vergence gradually decreases after the presentation of the cue stimulus. This decrease occurs because previous to the onset of the cue stimulus the angle of vergence increased by the presentation of both face images (the possible targets). Therefore at the time of the presentation of the cue stimulus eye vergence was returning towards baseline levels as we observed previously [18].

To determine whether there is a difference between the control group and ADHD group in eye vergence, we calculated the average size of the angle of vergence across both conditions (cue and no-cue) within the first 100ms after cue stimulus presentation as an indication of initial vergence angle and compared the values to the ones obtained from the final 100ms window of analysis, i.e. 900-1000ms after target presentation (see dark grey vertical bars in Fig 2). The results show a clear modulation (mean±sem: 0.021±0.0009) in the angle of vergence in the control group and a much weaker modulation (mean±sem: 0.011±0.001; t-test_56_ = 6.49, p<10^−8^) in the ADHD group.

**Fig 3:**
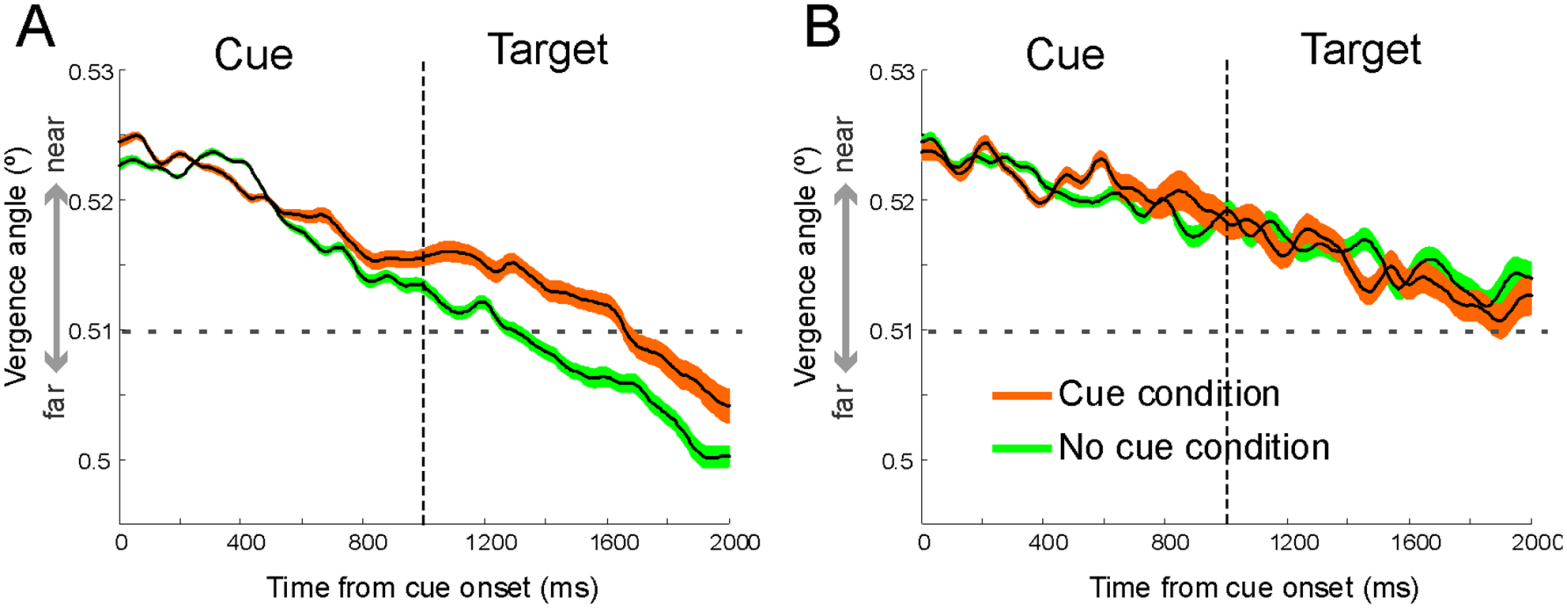
Average size of angle of eye vergence. Average (across all subjects) size of angle of vergence in the informative cue and uninformative cue conditions over time for the control group (**A**) and the ADHD group (**B**). Shaded areas represent ±1 times SEM around the mean. Dotted vertical lines indicate the time of target (face emotion) presentation. Horizontal dashed line indicates vergence angle belonging to the estimated screen distance.

We next analyzed vergence for the informative cue and uninformative cue condition separately (Fig 3). In the informative cue condition of the control group, the decrease in the size of the angle of vergence was weaker than the decrease in vergence in the uninformative cue condition. The differentiation started around 500ms after the onset of the cue stimulus (Fig 3A) and continued until the end of the trial (i.e. 1000ms after target presentation). The size difference in the angle of vergence between informative cue and uninformative cue condition was therefore most prevalent after target onset. In the ADHD group no difference in the mean angle of vergence between the informative cue and uninformative cue condition was observed (Fig 3B).

**Fig 4:**
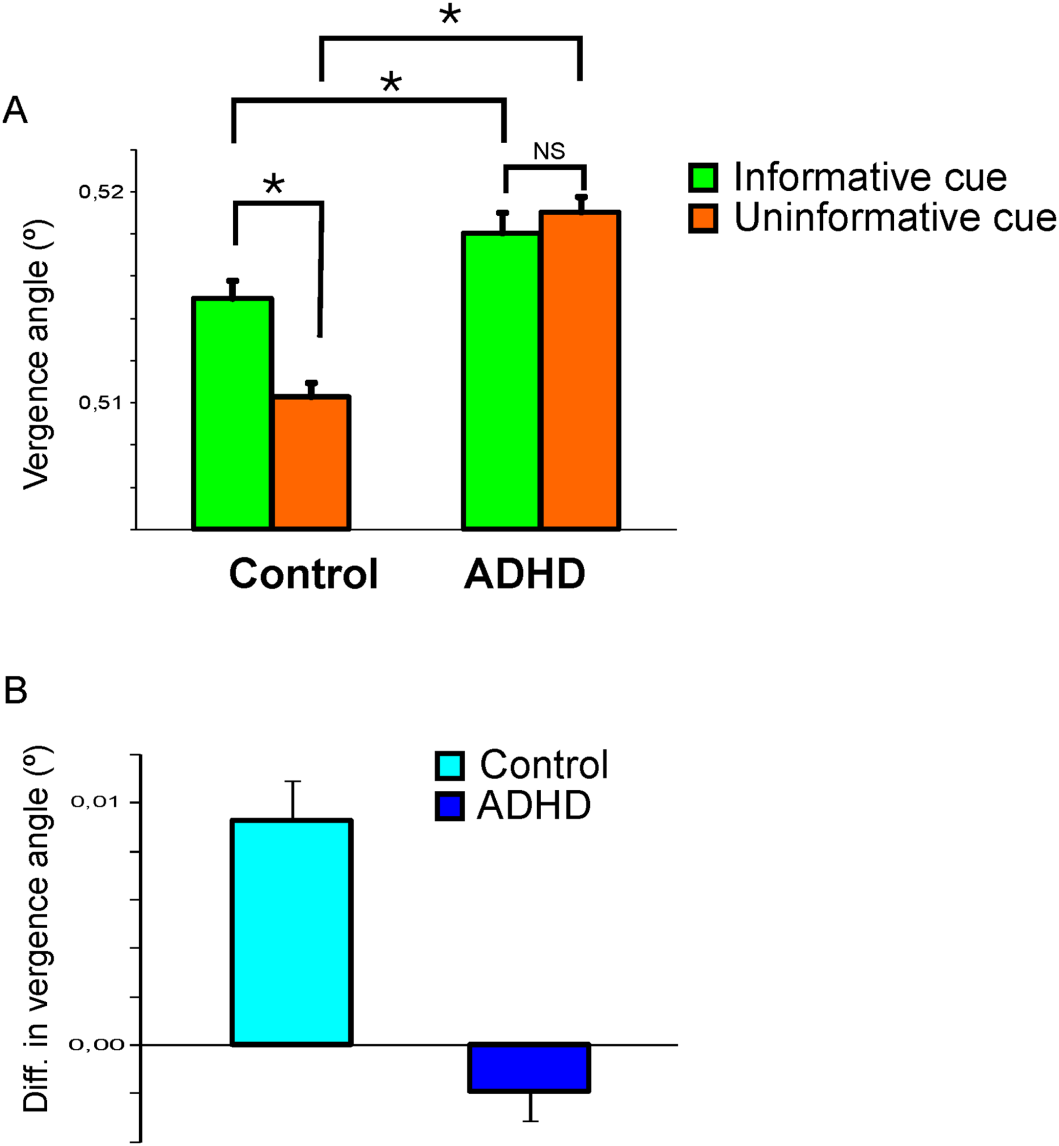
Mean sizes of angle of vergence for the different cue conditions (A) and the difference herein (B). Bars denote control/ADHD group. Error bars are SEM. Asterisks denote statistical significance. NS = not significant.

**Fig 5:**
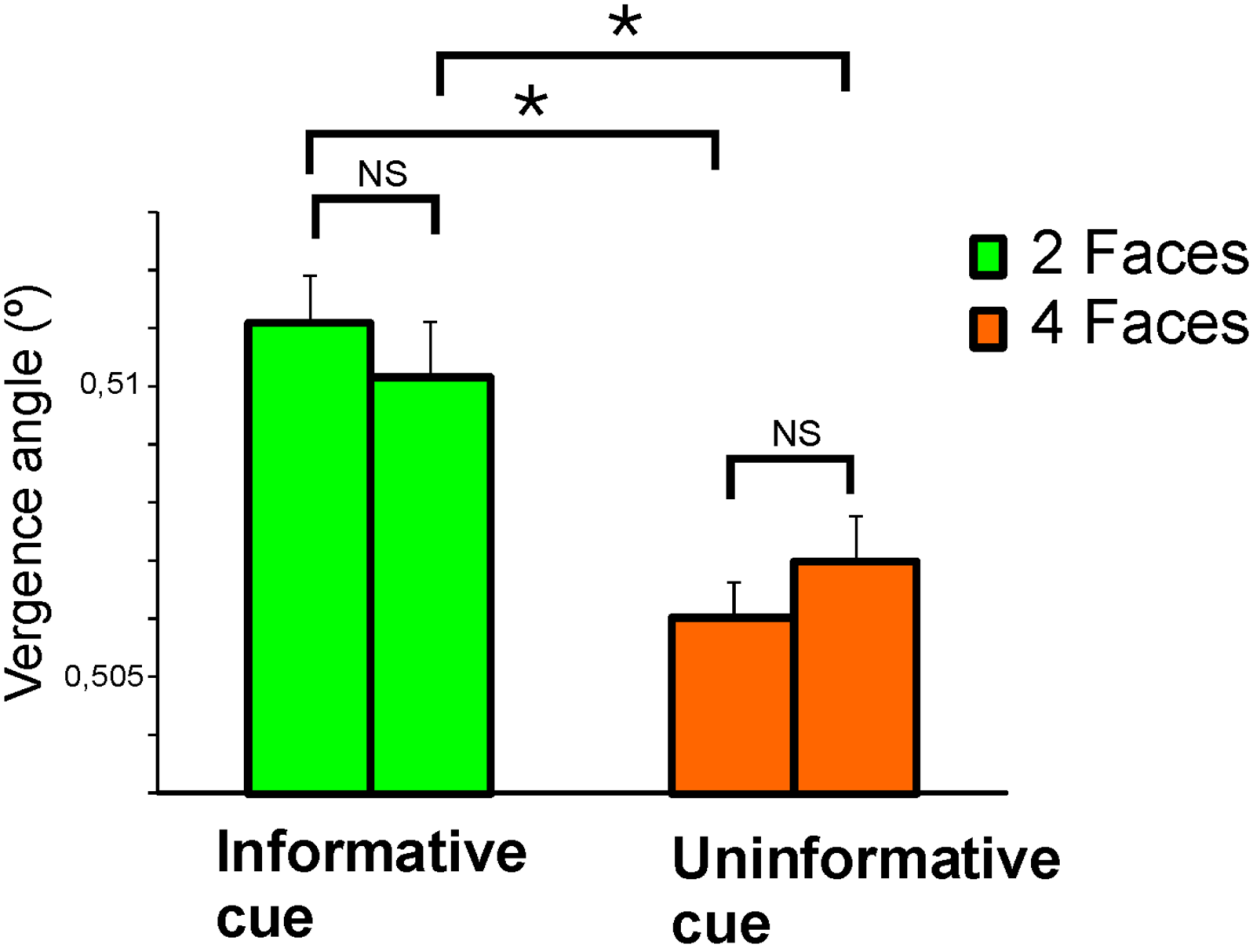
Mean sizes of angle of vergence for the different face conditions. Bars denote 2/4 face condition. Error bars are SEM. Asterisks denote statistical significance. NS = not significant.

To quantify these differences, we compared the mean strength of the modulation in the angle of vergence of the informative cue and uninformative cue conditions. For the calculation of the difference in vergence between the cue conditions, we selected a window of 1200 ms (800–2000 ms after cue onset; see light grey bar in Fig 2) to cover the period when the difference in the modulation in the angle of vergence between the informative cue and uninformative cue condition was most prominent. Before going on, a multi-way ANOVA test was applied in order to assess the interplay between the two existing factors, i.e., group (control, ADHD) and test condition (informative-uninformative cue). The outcome indicates a significant interaction between the two factors (F = 10.98, p = 0.001), in accordance with the observation that differences caused by trial condition are actually larger in the control group than in the ADHD group (see specific values below). The mean angle of vergence was different for the control group compared to the ADHD group (F_(1,57)_ = 31.5, p<10^−7^). In particular the mean angle of vergence in the uninformative cue condition of the control group was smaller than the mean angle of vergence in the uninformative cue condition of the ADHD group (F_(1,62)_ = 68.0, p<10^−11^; Fig 4A). We also analyzed the difference in angle of vergence between the informative cue and uninformative condition within groups. In the control group there was a significant difference between the informative cue and uninformative cue trials (Fig 4A and 4B; F_(1,68)_ = 19.3; p<10^−5^). In the ADHD group no difference in the mean angle of vergence between the informative cue and uninformative cue condition was observed (Fig 4A and 4B; F_(1,56)_ = 0.50; p = 0.47). We did not find a difference in vergence modulation between 2 face and 4 face tasks (Fig 5). Neither did we find significant differences in vergence between medicated and non-medicated ADHD patients.

**Fig 6:**
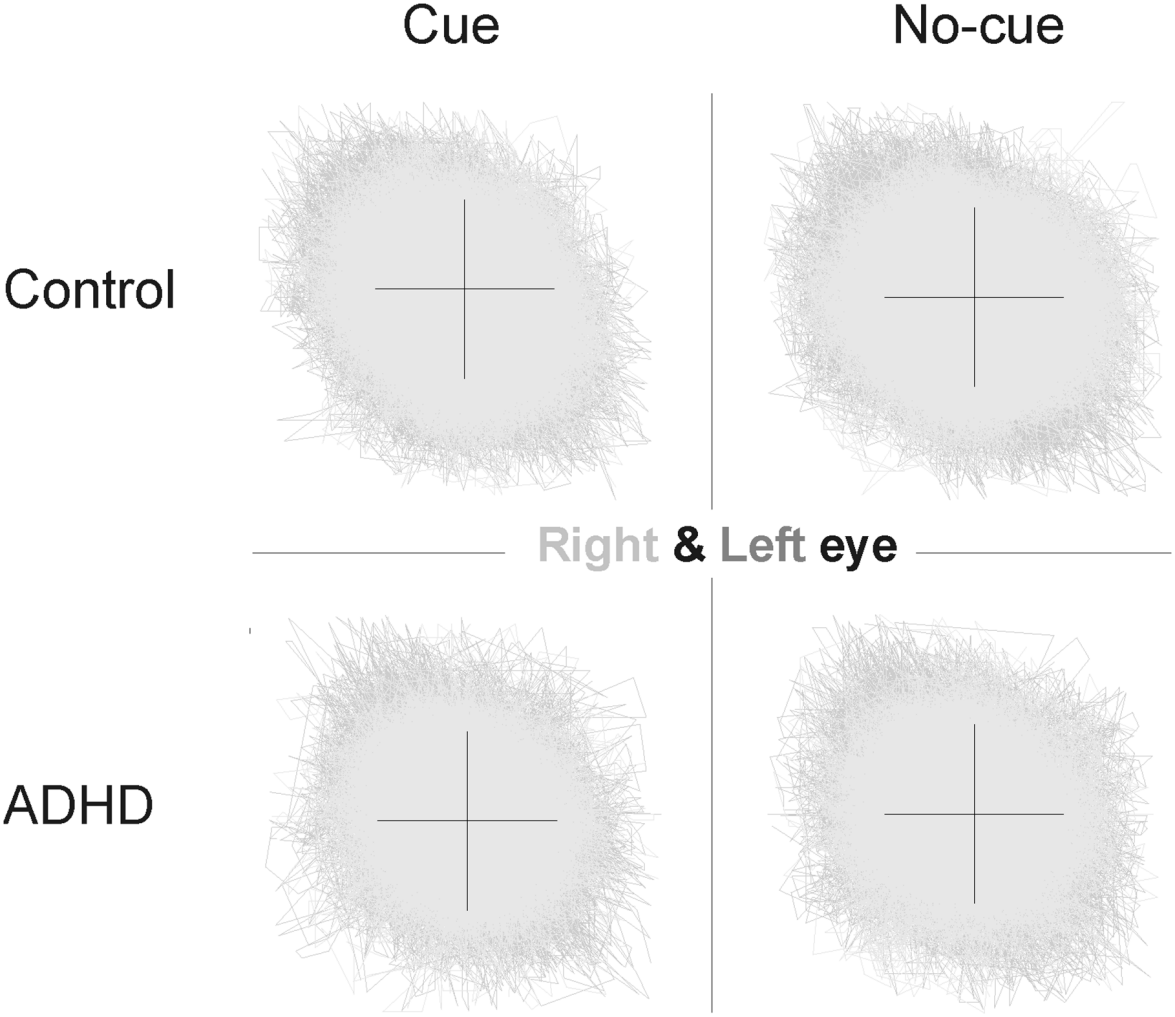
Eye positions of both eyes separately during the experiment. Traces represent the eye positions of time from cue stimulus onset until 1000ms after target presentation. Cross represents the approximate size of the fixation cross. Right eye and left eye positions are plotted on top of each other in different shadings of grey.

One of the symptoms of ADHD is a deficient oculomotor control [21,22], which may lead to more or larger eye movements. Although we removed trials that contained saccadic eye movements, we plotted the eye positions over time for both groups and conditions separately to confirm that gaze fixation during the trial was accurate. Fig 6 shows the eye traces of both eyes during the two seconds period after cue/no-cue presentation. Fixation micro-saccades uncover shifts of attention [6,7], and micro-saccades may, like target saccades result in divergence of the eyes during gaze fixation after a saccade. To analyze the variability in horizontal and vertical eye position during gaze fixation, we calculated the standard deviation (STD) of the eye position over time for both eyes separately. We found no significant (p>0.1) differences (STD: Control: Horizontal: informative cue, 5.175, uninformative cue, 4.890; Vertical: informative cue, 4.671, uninformative cue 4.652; STD: ADHD: Horizontal: informative cue, 5.295, uninformative cue, 5.119; Vertical, informative cue, 5.042, uninformative cue, 4,931) in variance. Thus these results are indicative of accurate gaze fixation, and support our previous findings showing that micro-saccades do not cause the difference in the angle of vergence between the cue and no-cue condition [18], and that noise levels in the eye recordings are similar for subjects tested at different locations (Hospital or school; Fig 6).

Children tend to have crossed disparity when fixation words [15], which may reflect the level of maturation of the vergence system. We estimated the difference in fixation disparity between Control and ADHD subjects by measuring the average vergence angle during the 100ms interval at the onset of the trial. During the initial fixation period both groups have similar (t-test_20_ = 0.05, p = 0.94) vergence angles at the start of the trial, which suggests no asymmetry in the direction of fixation disparity between Control and ADHD subjects.

## Discussion

Previously we demonstrated in adults that the angle of eye vergence changes during orienting visual attention [18]. In this study, we report that the relation between the modulation of the angle of vergence and covert attention also exists in children. In the control group, we observed a strong modulation in the angle of vergence and a difference between the cue conditions. In the ADHD group a weak modulation in the angle of vergence was observed and no difference between the cue conditions was noticed.

Compared to our first study in adults [18] the difference in vergence between the informative cue and uninformative cue condition is much weaker. This discrepancy may be explained by differences in age and/or by differences in task design. The task in [18] was to detect a minor change in a small stimulus whereas in the current study the size of the face stimulus and the change herein was quite large. Perhaps orienting attention was therefore less demanding resulting in weaker modulation in vergence. In both studies, we observed that the eyes converge after stimulus onset and that at the time of cue onset the eyes are still diverging; likely back to base line level (e.g. Fig 3C in [18]). Therefore the cue induced convergence is somewhat masked by this divergence. It is interesting to note that the eye divergence observed in the current study is more prolonged compared to the data in our earlier study [18]. This difference may be attributed to difference in maturity level of the vergence system (adults vs. children), to the applied stimuli (bars vs. faces), or to difference in the timing of stimulus presentation. Finally, our previous results [18] indicate that near-triad neither pupil size is an explanatory factor for the modulation of the angle of vergence. For instance, in our previous study [18] we tested the effect of target eccentricity on vergence modulation by presenting visual targets at a range of eccentricities. For all target locations, eye vergence was observed, and the angle was found to be similar across the eccentricities. We thus argue that target distance is not the main cause for the observed modulation of eye vergence.

We believe that eye vergence during gaze fixation is involved in orienting visuospatial attention [18]. This idea is agrees with a recent study showing a significant increase in fixation disparity for dyslexic children solely when reading [15,17]. These authors suggest that fixation disparity in dyslexics might be a result of the allocation of inadequate attentional and/or cognitive resources [15,23]. In conclusion our findings support the idea that fixation eye movements have a role in cognitive processing of sensory information. Besides attention, fixation eye movements may prevent loss of conscious vision [24], improve visual acuity [25,26], reduce binocular disparity [27], and adjust eye position after a target saccade [28].

### Function of attention related vergence

The role of the proposed attention related vergence is unknown but we propose [18] that the vergence modulation induces a switch in cortical state necessary for shifting attention to perceive visual stimuli [29–31]. Vergence is the opposite movement of both eyes, which is different than fixation and micro-saccades that are conjunctive eye movements, thereby changing disparity. Similar to micro-saccades that trigger neural responses in the visual cortex, the small attention related vergence may cause ERP responses in the parietal and occipital cortex [32]. It may thus be argued that during gaze fixation the small attention-related vergence specifically activates disparity cells at the foveal region. These cells have small receptive fields and are therefore most sensitive to small disparity changes. If we assume that vergence specifically activates central neurons, it may be argued that vergence has a role in the disengagement of attention. Cortical feedback that prominently targets cells located at the foveal region of the visual cortex [33] may control this process. We therefore suggest that the observed disruption in vergence in ADHD children reflects an inadequate cortical condition necessary for orienting attention.

Despite the difference in attention related vergence, in this study we did not find significant differences in behavior between the control and ADHD groups, although the ADHD group performed slightly worse on the task. Also, ADHD subjects responded faster in the informative cue condition than in the uninformative cue condition showing the behavioral improvement by attention. This seems at odds with the disruption of modulation of attention related eye vergence. This discrepancy can be accounted for by more variation in the ADHD subjects leading to a lack of significant difference between the different conditions. Large response variation is typically observed in ADHD patients and may lead to inconsistent findings in covert attention paradigms [34]. Alternatively, the task may have required only little cognitive effort, so that detection performance (which was around 90% for both groups) was not strongly dependent on attention recourses. According to this explanation vergence is not involved in visual detection but may relate to other cognitive processes being decision-making and perceptual awareness, which are not explicitly tested in this study. In conclusion, further research is needed to unravel the possible role of vergence in visual attention.

### Binocular vision and ADHD

Convergence insufficiency (CI) is a common binocular disorder characterized by the inability to obtain a single visual field while working at a near distance [35–38]. The primary source of CI symptoms may be accommodative insufficiency [39]. The adverse impact of CI occurs during near viewing where typical symptoms include double vision, blurred vision, eye strain, difficulty concentrating, and slow reading [36,37,40]. Many of the children with CI report attention problems [36,41] and recent studies demonstrate a relationship between CI and ADHD [19]. Our study supports the finding of binocular vision as an important factor in attention [42], and the idea of deficient binocular vision in ADHD children [19]. However, CI occurs at close distances whereas in our study the used target distances fall well outside the range of distances of CI for children. We therefore argue that our observed disruption in vergence modulation does not produce poor binocular vision per se, unlike the effects of CI in ADHD. For instance, detection performance in ADHD group was similar to the control group suggesting that visual discrimination was normal.

### Neurobiological implications

Top down attention originates in the frontal cortex [43,44] and reduced or distorted activation in prefrontal regions of ADHD patients has been observed [45–51]. Recent evidence shows that the frontal cortex controls eye vergence [52], which indicates that frontal region may be the source of our observed attention related vergence, and that a reduced functionality of the frontal cortex in patients with ADHD causes the disrupted modulation in attention related vergence. Furthermore, the frontal cortex is connected to the brainstem. Neurons in the brainstem, in particular in the midbrain reticular formation, control eye vergence [53–58]. The reticular formation forms part of a broader pathway, including the frontal and parietal regions of the cerebral cortex [59–61] and cerebellum [61–63] that is involved in the control of vergence eye movements. It is thus not unreasonable to assume that the brainstem nuclei involved in vergence control also form part of the attention system of the brain. For instance, using neuro-imaging techniques a recent study reported brainstem abnormalities in ADHD patients and showed 93% prediction accuracy [64].

### Neurodevelopmental relevance

Ocular dominance columns in the visual cortex are formed during early ontogenetic stages and can be modified after birth during the sensitive period. Recent evidence shows that ocular dominance maps may serve as a scaffold for the formation of disparity maps [65]. Disparity neurons in the primary visual cortex can detect the existence of disparity in their input from the eyes. The vergence observed in attention tasks implies that retinal disparity changes during shifts of attention, thereby activating disparity neurons in the visual cortex. Accordingly, in addition to their involvement in depth perception disparity neurons also may participate in the attention system in the brain. This notion is supported by findings of a disconnection between vergence and depth perception [66,67] and by a specialization of horizontal disparity in the primate primary visual cortex [68]. Also recent research suggests that in addition to primary visual loss, people with amblyopia—a loss of acuity in one eye following abnormal binocular experience during an early critical period—may also have deficient attention mechanisms [69]. Thus, if true then our findings suggest that precise cortical developmental organization, i.e. correct axonal termination patterns and neuronal positioning into ocular columns [70], is beneficial for subsequent attention processing of incoming sensory information. Incorrect development of ocular dominance maps may then result in an attention deficit, as in ADHD.

### Clinical relevance

Our observations show that attention related vergence differs between controls and children with ADHD. This finding points to the possibility that evaluation of vergence during an attention task may be useful for the development of an observer independent tool for the diagnosis of ADHD. Furthermore, for people with binocular deficits, vision therapy increases the eyes' convergence ability with eye-focusing exercises. Typically, these exercises include simple ‘pencil pushups’, computer vision therapy, or glasses with built-in prisms. It will be interesting to examine whether attention related vergence can be improved by vision therapy that employs purposed designed visual tests.

### Study limitation

This is the first study assessing attention related vergence in children with ADHD. The study used a limited number of subjects and children were recruited differently; ADHD patients from a hospital and controls from a public school. Although the ADHD group was sex/age matched with peers, recruiting children differently may have introduced a discrepancy in educational and social level between both groups resulting in a difference in attention-related vergence. Moreover, children were tested in different rooms that may have different lighting condition and may affect the quality of the eye recording data, although lightning conditions were kept similar as possible. If our results were attributed to differences in lightning conditions, we would have seen global differences in the recordings and not in a specific stimulus locked window. Moreover the calibrations qualities were similar in both conditions indicating that differences in noise level in eye position data cannot explain the observed difference in eye vergence (Fig 6). The outcomes of the analysis of eye positions during central fixation support this conclusion. The training with 10–20 practice trials can neither account for the results. Detection performance was the same in both groups demonstrating that children from both groups performed equally well on the task. Also overall performance was very high (90%) suggesting that the task was easy. Finally, further studies are needed to investigate whether medication has an effect on the attention-related vergence and whether attention-related vergence differs among presentation “specifiers” (subtypes).

## Conclusions

Our study supports the observation of deficient binocular vision in ADHD patients. We argue that the observed disruption in vergence eye modulation in children with ADHD is manifest of a deficient cognitive processing of sensory information. Our work may provide new insights into the neurobiology of ADHD, and may be the start of developing new and complementary tools for probing ADHD. Further studies are needed to resolve a possible role of eye vergence in visual cognition.
